# Surgical Experiences of Patients From the Circumpolar North: A Scoping Review

**DOI:** 10.1002/wjs.70034

**Published:** 2025-08-06

**Authors:** Jillian Schneidman, Sara B. A. Morel, Vanessa Ross, Natasha G. Caminsky, Jeremy Grushka, Evan G. Wong

**Affiliations:** ^1^ Faculty of Medicine and Health Sciences McGill University Montreal Quebec Canada; ^2^ Division of Trauma and General Surgery Department of Surgery McGill University Health Centre Montreal Quebec Canada

**Keywords:** circumpolar north, patient experience, scoping review, surgery

## Abstract

**Background:**

The Circumpolar North is an expansive region that includes the northernmost parts of the Earth, extending across the Arctic and Subarctic zones. This area, characterized by its geographical remoteness, harsh climate, limited healthcare infrastructure, and diverse patient population, creates unique challenges for the provision and delivery of surgical care. Despite existing research in this field, there is a lack of comprehensive reviews summarizing patients' distinct experiences when accessing surgical care. This scoping review, therefore, aimed to fill this gap by mapping the current literature on the surgical experiences of patients from the Circumpolar North.

**Methods:**

A scoping review methodology was employed to identify relevant, original, peer‐reviewed articles across seven databases, with no limits on publication dates. Article screening and data extraction were undertaken independently by two reviewers. An iterative data analysis process was employed to categorize findings from the included studies and identify common patterns, key insights, and knowledge gaps.

**Results:**

A total of 17 studies were included in this review. Key factors influencing the surgical care experiences of patients from the Circumpolar North were identified across four domains: (1) logistical factors, including proximity to care centers, temporary accommodations, and financial costs; (2) psychosocial factors, such as experience of medical evacuations, separation from family, and reintegration into home communities; (3) cultural factors, encompassing navigating healthcare environments, language differences, and nonverbal communication; and (4) medical factors, including patient involvement, healthcare provider interactions, and continuity of care. Several studies also highlighted patients’ experiences regarding innovative models aimed at improving locally based surgical care, such as telehealth and community‐based strategies.

**Conclusion:**

This review summarized the literature on the surgical care experiences of patients from the Circumpolar North. It offers insights into improving healthcare interactions and systems to better serve this population. It also highlights significant research gaps, particularly regarding Indigenous patient experiences and the impact of medical evacuations across diverse surgical specialties. Addressing these gaps through future research is crucial for deepening our understanding of surgical experiences of patients from the Circumpolar North and developing more effective, culturally competent strategies to improve patient care.

## Graphical abstract

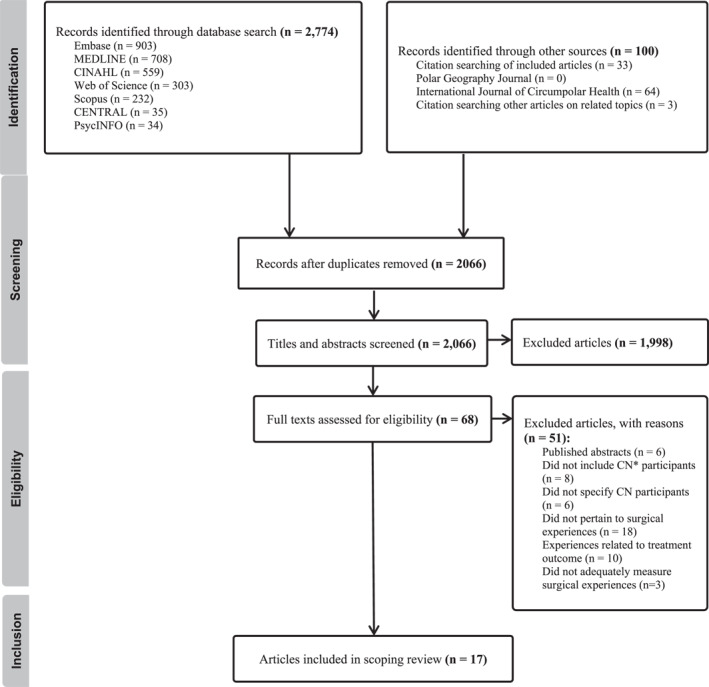

## 1 Introduction

The Circumpolar North comprises a vast and loosely defined region centered around the North Pole, spanning the Arctic and Subarctic zones. Typically depicted on a polar projection map, it extends from 55°N to 90°N latitude and includes portions of eight countries, referred to as the *Arctic Eight*: Norway, Sweden, Finland, Russia, the United States, Canada, Denmark, and Iceland [1].

Despite their unique landscapes, regions within the Circumpolar North share similar geographical and systemic characteristics that present significant obstacles in delivering high‐quality surgical care. Many cover vast geographic areas but are home to small, widely dispersed populations, generally with fewer than five people per square kilometer [2]. Such low population density makes it difficult to develop and sustain comprehensive healthcare infrastructure, particularly surgical services, leaving many residents with limited access to surgical care [3, 4, 5]. As a result, patients often travel long distances to reach care, either relying on a few centralized regional centers or distant tertiary care institutions for specialized or emergency services [4]. This travel is further complicated by restricted access routes, with patients frequently depending on air transport or seasonal travel methods—both of which are highly vulnerable to extreme and unpredictable weather conditions [4, 6]. Together, these factors can lead to significant delays in accessing and receiving surgical care, heightening health risks and worsening patient outcomes [7, 8].

In addition to these logistical hurdles, patients from the Circumpolar North, particularly patients from Indigenous communities, face significant cultural and social challenges when seeking surgical care far from home. Their journeys to healthcare facilities often bring them into unfamiliar environments and settings, where they may encounter language barriers, differing cultural norms, and instances of discrimination [4, 9, 10]. For Indigenous populations, mistrust of the healthcare system, stemming from past negative experiences and systemic marginalization, can add layers of complexity to these interactions with healthcare services [11, 12, 13].

Although research on patients from the Circumpolar North has grown in recent years [14], no reviews to‐date have summarized patients' experiences with surgical care. Existing reviews have either focused on specific aspects of surgical care, such as surgical outcomes [15], or have taken a broader perspective by exploring general patient experiences [4, 6, 16]. Addressing this critical gap is essential for gaining valuable insights that can enhance the provision and delivery of surgical care, inform targeted interventions, promote culturally sensitive practices, and identify areas requiring further exploration in surgery. A scoping review is particularly well‐suited, as it allows for the examination of a diverse topic, providing an overview of the existing literature without the constraints of narrowly defined research questions typical of systematic reviews [17].

## 2 Methods

This scoping review was conducted using the six‐step methodology outlined by Arksey and O’Malley [17], and adhered to the Preferred Reporting Items for Systematic Reviews and Meta‐Analyses (PRISMA) extension for Scoping Reviews [18]. The protocol for this scoping review is registered and available online [19].

### 2.1 Search Strategy

The search terms for this review were developed by building on previous literature reviews of related topics and consulting with a research librarian. Our aim was to answer the following question: What are the surgical experiences of patients from the Circumpolar North? To address this, the search terms were centered around three concepts: patient experiences, surgery, and Circumpolar North. The Circumpolar North was defined geographically as the regions of the Arctic Eight that span from 55°N to 90°N latitude. The keywords used for the database searches are reported in Table 1.

**TABLE 1:** Keywords for database searches to identify studies on surgical care experiences of patients from the Circumpolar North.

Concept	Keywords
Patient experiences	Exp qualitative research/OR interviews as topic/or narration/or self‐report/or focus groups/or patient reported outcomes measures/or “surveys and questionnaires”/OR (qualitative* or survey* or interview* or focus* group*).tw,kf. OR (“content analysis” or descriptive or discourse* or ethno* or “grounded theory” or interpretive or “mixed method*” or narrative or phenomenolog* or thematic* or theme*).tw,kf.
Surgery	Exp surgery/OR (surgery* or surgical* or surgeon*).tw,kf. OR exp surgical procedures/
Circumpolar north	Inuit/or Nunavut/or Northwest Territories/or Yukon territory/or Alaska/or Alaska natives/or Greenland/or Faroese people/or Iceland/OR [Canada/or Quebec/or Manitoba/or “newfoundland and labrador”/or Americas/or Russia/or siberia/or Norway/or Finland/or Sweden/AND (north* or arctic or polar or circumpolar).tw,kf.] OR (arctic* or subarctic* or inuit* or yupik* or yup'Ik* or innu* or alutiiq* or kalaallit* or Inupiat* or eskimo* or eskaleut* or “canad* north*” or “north* canad*” or inuvialuit* or “northern Quebec” or nunavik* or nunavummiut* or nunatsiavut* or nunatukavut* or Nunavut* or qikiqtaaluk* or “northwest* territories*” or yellowknife* or Yukon* or Alaska* or “Alaska* native*” or Greenland* or “faroe islands” or chukchi* or “chukchi peninsula” or koryaks* or chukotka* or “northwest Russia*” or “northern* siberia*” or Sami or “north* Norway*” or “northern Finland” or finmark or lappi or “north* Sweden*” or Iceland*).

An extensive search was conducted in July 2024 across seven databases, with no restrictions on publication dates. The databases searched were: Embase, MEDLINE, CINAHL, Web of Science, Scopus, CENTRAL, PsychINFO. Additionally, hand searches were performed across Polar Geography, the International Journal of Circumpolar Health, reference lists of included studies, and reference lists of other articles on related topics. Articles were imported into EndNote and were uploaded to the Covidence Software for removal of duplicates, title and abstract screening, full‐text review, and data extraction.

### 2.2 Study Selection

Titles and abstracts were screened by two independent reviewers (JS, VR) based on predefined eligibility criteria. Following this, full‐text screening of the remaining articles was conducted by two independent reviewers (JS, SM). At both stages, any disagreements were resolved between the two reviewers, with the involvement of an independent third reviewer (NC) if a consensus could not be reached.

Studies were included if they met the following criteria: (1) original, peer‐reviewed research, (2) focused on patients from the Circumpolar North, (3) assessed patient experiences (i.e., satisfaction, perspectives, opinions, views, perceptions, and/or responses) with surgical care, and (4) published in English. Additionally, studies were included that involved populations who, although not explicitly specified as having received surgical care, could require surgical interventions at any point (e.g. high‐risk births). Articles were excluded if they: (1) were study protocols, case reports, or presentations/conference abstracts; (2) included multiple populations without separate data on patients from the Circumpolar North; and (3) focused solely on specific surgical outcomes of the surgery itself rather than the overall care experience.

### 2.3 Data Extraction and Analysis

Data collection and extraction from each included article were conducted independently by two reviewers (JS, SM). Information was extracted from each study into a standardized Microsoft Excel (version 16.95.3) spreadsheet and included: citation, study objective, study design, study location, study care setting, surgical specialty, participant characteristics, and main study findings and recommendations. A quality assessment was omitted, consistent with the aim of scoping reviews to map existing literature rather than assess study quality [17].

An iterative data analysis process was employed to uncover common patterns and key insights from the included studies. The findings from the articles were initially coded independently by JS and SM. Following this, the codes were compared, discussed, and refined to identify overarching categories. These categories were then reviewed and revised through discussions with additional team members (NC and EW).

## 3 Results

**FIGURE 1:**
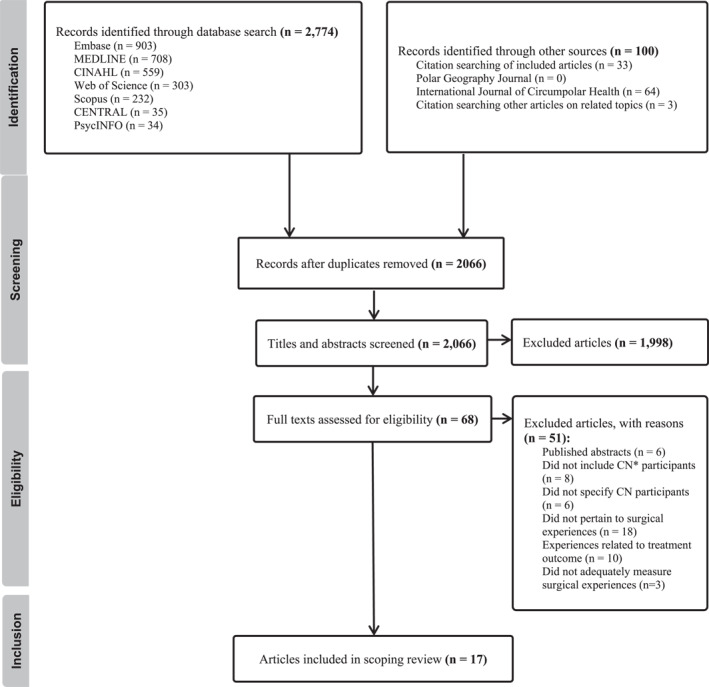
PRISMA flow diagram of the selection process for studies evaluating the surgical experiences of patients from the Circumpolar North. CN: Circumpolar North.

Figure 1 illustrates the flow of studies throughout the review process. A total of 17 papers were included. Table 2 provides a summary of the included studies. All Circumpolar North countries were represented in at least one article, except Russia. Canada had the highest representation of studies (*n* = 7), followed by Norway (*n* = 4) and Finland (*n* = 2). Most articles focused on obstetrics and gynecology (*n* = 5), general surgery (*n* = 5), and orthopedic surgery (*n* = 4). Six studies focused on inpatient care, while 11 studies took place in outpatient care settings.

**TABLE 2:** Overview of included studies in scoping review.

Author and year	Research objective	Data collection tool	Location	Care setting	Surgical specialty	% CN^a^ participants	% indigenous participants
Aarnio et al., [20]	To evaluate user satisfaction with surgical teleconsultation.	Questionnaire	Finland	Outpatient	General surgery, orthopedic surgery	100%	Not specified
Buvik et al., [21]	To compare patient‐reported health outcomes and satisfaction between video‐assisted remote and standard face‐to‐face orthopedic consultations.	Questionnaire	Norway	Outpatient	Orthopedic surgery	100%	Not specified
Cano and foster, [22]	To better understand women's experiences seeking and obtaining abortion care.	Semi‐structured interviews	Yukon territory	Outpatient	Obstetrics and gynecology	100%	Not specified
Chamberlain and barclay, [23]	To describe the psychosocial effect of requiring canadian inuit women to transfer out of their communities for birth.	Semi‐structured interviews	Central arctic region in Canada	Inpatient	Obstetrics and gynecology	100%	100%
Chan et al., [24]	To evaluate patient satisfaction and surgical outcomes with a rural obesity program.	Questionnaire	Alberta and Yukon territory	Outpatient	General surgery	50%	Not specified
Haukipuro et al., [25]	To analyze the feasibility of videoconferencing in the examination of orthopedic outpatients.	Questionnaire	Finland	Outpatient	Orthopedic surgery	100%	Not specified
Holtan, [26]	To analyze patients experiences with telemedical consultations between a general practitioner and a specialist in otolaryngology.	Unstructured interviews	Norway	Outpatient	Otolaryngology	100%	Not specified
Irvine et al., [27]	To explore the perspectives and preferences of pregnant women receiving prenatal care in a rural community regarding delivery location.	Semi‐structured interviews	Saskatchewan	Inpatient	Obstetrics and gynecology	100%	Not specified
Krane et al., [28]	To explore patients’ experiences of aspects contributing to safety and in healthcare services following percutaneous coronary intervention.	Semi‐structured interviews	Norway	Outpatient	Cardiac surgery	100%	Not specified
Naverlo, gunnarsson, and strigard, [29]	To investigate the impact of distance to nearest hospital on the quality of life of rectal cancer patients who receive a stoma at index surgery.	Questionnaire	Sweden	Outpatient	General surgery	100%	Not specified
Pedersen and holand, [30]	To determine whether patients are satisfied with telemedicine consultations compared to face‐to‐face consultations for otorhinolaryngological examinations.	Questionnaire	Norway	Outpatient	Otolaryngology	100%	Not specified
Seibæk, [31]	To explore patient perspectives on drivers and barriers to patient involvement in Greenlandic hospital care.	Semi‐structured interviews, participant observations	Greenland	Inpatient	Not specified	100%	Not specified
Sigurðardottir, [32]	To investigate the level of satisfaction of care received among patients undergoing ambulatory surgery in two hospitals in Iceland.	Questionnaire	Iceland	Inpatient	General surgery, orthopedic surgery, urology, vascular surgery	100%	Not specified
Silver et al., [33]	To examine how best to support culturally safe birth for inuit families when birth must take place away from home.	Fuzzy cognitive mapping	Canada	Inpatient	Obstetrics and gynecology	100%	24%
Smith et al., [34]	To understand the trends and reasons for colonoscopy cancellation in Northwest Territories.	Chart review	Northwest Territories	Outpatient	General surgery	100%	Not specified
Vang et al., [35]	To document and analyze patient‐provider encounters from the perspectives of indigenous women with medically high‐risk pregnancies who were transferred or medevacked to tertiary care centers.	Semi‐structured interviews	Quebec	Inpatient	Obstetrics and gynecology	100%	100%
Wetterhall et al., [36]	To evaluate the effectiveness and impact of the dental health aide therapist model in providing dental care to remote villages.	Semi‐structured interviews, direct observation, questionnaire	Alaska	Outpatient	Surgical dentistry	100%	Not specified

^a^ CN: Circumpolar North.

### 3.1 Factors Influencing Surgical Care Experiences

Ten articles explored the conventional surgical care experiences of patients from the Circumpolar North [22, 23, 27, 28, 29, 31, 32, 33, 34, 35]. Among these studies, a range of factors shaped these experiences, which were categorized into four areas: logistical, psychosocial, cultural, and medical (see Table 3 for a summary of the description of these factors).

**TABLE 3:** Factors from included studies that influenced the surgical experiences of patients from the Circumpolar North.

Factor	Description
Logistical factors	
Proximity to care centers	Distance patients travel to access surgical care centers.
Temporary accommodations	Availability and quality of short‐term accommodations near surgical care centers.
Financial costs	Costs related to surgical care, including travel expenses, accommodations, additional costs for family members.
Psychosocial factors	
Experience of medical evacuations	Impact of medical evacuations on patients' psychological well‐being.
Separation from family	Duration, extent, and psychological effects of separation from loved ones during surgical care.
Reintegration into home communities	Process and ease of reintegration back into daily life following surgical care.
Cultural factors	
Navigating healthcare environments	Ability to adjust and adapt to unfamiliar healthcare environments.
Language differences	Ability to verbally communicate with surgical care providers, including having access to interpreters.
Nonverbal communication	Impact of gestures, body language, and other nonverbal actions when interacting with surgical care teams.
Medical factors	
Patient involvement	Extent of patient participation in surgical decision‐making and treatment choices.
Healthcare provider interactions	Experiences and relationships with surgical care providers.
Continuity and coordination of care	Consistency of surgical care across different settings, providers, and stages of the care pathway.

### 3.2 Logistical Factors

Nine articles highlighted logistical challenges affecting the surgical care experiences of patients from the Circumpolar North [22, 23, 27, 28, 29, 32, 33, 34, 35]. Proximity to care centers emerged as a key factor, with patients living closer to hospitals—especially those that offered specialized or emergency care—reporting greater reassurance and more positive care experiences [27, 28]. In contrast, patients residing further away experienced heightened stress due to long travel times, limited access to public transportation, and seasonal weather conditions, particularly during winter months [28]. These logistical challenges also resulted in frequent delays in care, cancellations of appointments, and worse surgical outcome experiences, such as heightened pain or worsened quality of life following surgery [22, 28, 29, 34].

Several articles also discussed how temporary accommodations near healthcare facilities influenced patients' surgical care experiences [33, 34]. For those traveling long distances to surgical appointments, coordination between multiple agencies (e.g., those managing medical travel, accommodations, and scheduling) often led to miscommunications about housing availability and suitability, leaving some patients without accommodations, forcing them to cancel appointments [34]. When housing was found, many reported that it was often unsuitable for extended stays due to small rooms, uncomfortable beds, limited internet access, and security concerns—all of which added stress to an already challenging experience [33].

Financial difficulties were also commonly discussed across studies, with transportation costs, including taxi fares or fuel for long drives, proving burdensome for patients requiring multiple trips to surgical healthcare facilities [22]. For patients who were emergently evacuated by airplane, the financial strain increased when they wished to bring more family members than the healthcare facility allowed (typically one escort) [33]. Covering this airfare for additional companions was often prohibitively expensive, leaving many patients without the crucial emotional and practical support networks that could have improved their care experiences [33].

### 3.3 Psychosocial Factors

Six studies reported on psychosocial factors [23, 27, 28, 31, 33, 35]. A common theme was the psychological impact of emergency evacuations, particularly for high‐risk surgical cases, as patients were often transferred abruptly to distant hospitals with little warning, facing significant uncertainty and distress [23, 27, 33, 35]. For many, this represented more than just a medical event—it was a major transition that separated patients from their familiar surroundings, family, culture, and social support networks [23, 27].

While in the hospital, being physically separated from family emerged as a key factor shaping patient experiences. Patients who were separated from their families frequently reported feelings of loneliness and isolation, particularly during critical moments of care and recovery [23, 27, 31]. This separation was especially difficult for mothers, who expressed concerns about the well‐being of children left behind as caregiving responsibilities shifted to husbands or extended family members [23, 35]. Efforts to maintain family involvement, such as bringing relatives to the hospital, were reported to positively impact patients’ overall care experiences [28]. However, such efforts were not always possible due financial, logistical, or policy‐related barriers [33, 35].

Separation during hospitalization not only affected patients but also placed significant strain on their families. Physical distance and limited communication often left families uncertain about the patient’s condition and overall health, creating additional stress [23, 31]. This lack of information had lasting effects after discharge, with families struggling to support patients without a clear understanding of their physical and emotional needs [31]. For mothers returning home after childbirth, separation sometimes disrupted family dynamics, as young children associated their mother’s absence with the arrival of the newborn [23]. Conversely, when family members were present during hospital stays or when patients were allowed to give birth within their communities, post‐hospitalization support improved and family connections were strengthened [23].

### 3.4 Cultural Factors

Five articles explored cultural factors that influenced the surgical care experiences of patients from the Circumpolar North [23, 28, 31, 33, 35]. A major challenge identified was patients’ difficulty in navigating unfamiliar healthcare environments, particularly those that were highly complex, such as tertiary care settings [23, 27]. Moreover, larger hospitals, with longer wait times, brief medical visits, and repeated interactions with multiple providers asking for the same information, intensified patients’ confusion and frustration and contributed to feelings of disconnection and impersonality in their care [28, 31, 33, 35].

Language differences also significantly impacted patient experiences, as it made it more challenging for patients to fully understand medical information, the consent process, and discharge instructions [28, 33]. The presence of a family member or interpreter from the same linguistic background helped bridge these gaps by facilitating communication in the patient’s native language [35]. These individuals also often shared similar cultural values and practices, making patients feel more at ease in the healthcare setting [23, 27]. For hospitalized patients, this additional cultural support was especially important, as many reported a lack of culturally appropriate food options and social activities that met their needs [33].

Non‐verbal interactions also affected patients. Differences in communication styles, such as eye contact, body language, and gestures at times led to misunderstandings between patients and healthcare providers [35]. For example, quieter or more reserved patients were sometimes misinterpreted as rude or avoidant by their provider, creating tension in their relationship [35]. These challenges were further compounded by racial stereotypes about Indigenous patients, including assumptions of noncompliance or lack of education, leading providers to dismiss them and wrongly assume they were unable to understand medical instructions [33]. Instances of overt racism, such as security guards searching patients’ pockets based on appearance, intensified these feelings of judgment and led many patients to withdraw physically or emotionally from their care [33].

### 3.5 Medical Factors

Seven articles examined medical factors that influenced surgical care experiences [22, 23, 27, 28, 31, 33, 35]. Many patients expressed the importance of patient engagement, with a strong desire to be involved in decisions about their care [35]. However, patients often felt excluded as healthcare professionals made decisions without their input, either due to communication barriers or a lack of thorough discussions on the risks and benefits of care plans [23, 31, 33]. Additionally, many patients reported that their cultural practices were often overlooked during their treatment, which limited their autonomy in their care and reinforced their sense of being “guests” in a foreign environment [23, 33].

Another critical factor shaping patients’ surgical experiences was their relationship with healthcare providers. Many patients felt neglected when providers focused solely on their routine tasks, interpreting this as a lack of empathy and engagement during a time when patients felt especially vulnerable [23]. In contrast, when providers did offer more personalized care (e.g., addressing them by their first name, acknowledging patients' emotional states), it helped build trust and eased the difficulties patients were having in other aspects such as language or feelings of disempowerment [31].

Patients also highlighted that consistency and continuity of surgical care significantly influenced their experiences. In rural areas, reliance on locum general practitioners and high staff turnover created a sense of instability, raising concerns about care quality and deterring patients from seeking treatment [27, 35]. Furthermore, the lack of local specialists often forced patients to schedule multiple appointments with different providers, leading to frustration [22]. For those recovering from surgery, inconsistent follow‐up care, long travel distances, difficulty understanding discharge instructions, and a lack of trust in local providers further complicated patients’ recovery process [35].

### 3.6 Innovative Models to Enhance Surgical Care Experiences

Seven articles examined innovative models aimed at improving surgical care experiences for patients from the Circumpolar North [20, 21, 24, 25, 26, 30, 36]. These included telehealth and community‐based care interventions.

### 3.7 Telehealth Models

Telehealth models primarily used videoconferencing for surgical consultations to enable patients to consult with surgical specialists while remaining within their local communities. In some cases, patients underwent physical examinations conducted by a general practitioner [20, 25, 26] or a trained nurse [21] in a local health center, with a surgeon observing via video. In other models, trained general practitioners performed physical examinations and procedures such as endoscopy in a local health center, and then shared the findings to a surgical specialist who had a one‐on‐one video consultation with the patient [30].

Patients generally reported positive experiences with telehealth, finding it to be a convenient and effective alternative to in‐person hospital visits, and expressing high satisfaction with the care received [20, 21, 25, 30]. Most felt telemedicine did not compromise the quality of care [30] and were more likely to choose videoconferencing for future surgical visits [21, 25, 30]. However, experiences varied depending on technological factors, with clear audio and imaging being essential for effective consultations [20, 25]. Patients also valued consultations that involved both a general practitioner and a specialist, as they believed it improved diagnostic accuracy [20, 26].

### 3.8 Community‐Based Care Models

Community‐based care models aimed to enhance access to local services and specialists within patients’ local communities. One model entailed training mid‐level providers to deliver essential surgical care within the local community [36], while another involved bringing surgical teams from tertiary centers to the community on specific days to perform pre‐ and post‐operative consultations [24].

Overall, these models were well received, with patient satisfaction comparable to conventional care [24, 36]. These models enhanced access to preventive and minor surgical care, strengthened patient‐provider relationships, and improved follow‐up experiences by making post‐treatment assessments more accessible [24]. However, complex and urgent cases still required referral to tertiary centers, where patients faced the usual challenges with conventional care experiences [24, 36].

## 4 Discussion

This scoping review explored the surgical care experiences of individuals from the Circumpolar North, highlighting the diverse logistical, psychosocial, cultural, and medical factors that shape patients’ experiences. It also examined the experiences of innovative surgical strategies aimed at improving local care, particularly for less urgent needs or when the physical presence of a specialized surgeon is not required (e.g., during certain pre‐surgical or post‐surgical visits).

This review highlights the critical role healthcare professionals play in improving the surgical care experiences of patients from the Circumpolar North. It emphasizes the need for providers to develop cultural competence to address the unique challenges faced by patients from this region, particularly Indigenous patients, who continue to experience the historical and ongoing impacts of colonization. Additionally, this review reinforces the importance of effective, clear, and accessible communication in building trust and ensuring patients feel informed and involved in their care decisions. These findings align with those reported in other scoping reviews focused on healthcare delivery to patients from the Circumpolar North [4, 6].

At the systemic level, this review underscores the need for structural changes to overcome barriers faced by patients from the Circumpolar North. Expanding surgical care infrastructure and improving access within regional healthcare centers could reduce reliance on medical evacuations and provide more timely care options closer to home [37]. Public health strategies should also focus on alleviating the emotional burdens of evacuations by supporting family and cultural connections. This could include transportation policies allowing multiple escorts or creating support networks for patients far from their communities. Peer navigation programs, that have been proven to be successful in other healthcare contexts [38], could be a viable option to guide patients through their surgical journeys, offering emotional, practical, and cultural support. Furthermore, collaboration between local and specialized healthcare providers is crucial for ensuring continuity of care and improving surgical outcomes, particularly after discharge. Innovations such as telemedicine, combined with local healthcare workers' involvement in pre‐ and post‐surgical care, can bridge service gaps, reduce reliance on distant specialists, and offer a more sustainable, accessible model of care, as demonstrated by several studies included in this review [20, 21, 25, 30].

Although patients in nonurban regions share common barriers to surgical care—such as limited resources, geographical isolation, and cultural factors [39, 40, 41]—the Circumpolar North presents a distinct set of challenges. The region’s vast geography, extreme weather, and isolation severely complicate patients’ access to surgical and health services [6]. Unlike other rural areas, where regional hospitals are often located closer to patients and can help mitigate some barriers through localized and specialized care [40, 42], the Circumpolar North often lacks such infrastructure. This leads to longer travel times, delays in care, more frequent medical evacuations, diminished continuity of care, and extended separations from family, all of which significantly impact patients throughout their surgical journeys, as evident throughout this review. These realities emphasize the need for tailored strategies that address the unique environmental, infrastructural, and logistical barriers faced by patients from the Circumpolar North.

The findings from this scoping review are significant not only for the insights they provide but also for the gaps that they reveal. One key gap in the current literature is the underrepresentation of studies specifically examining Indigenous patients' experiences. Many existing studies do not specify the proportion of the population that is Indigenous versus non‐Indigenous, making it difficult to determine the extent to which Indigenous perspectives are reflected. This limitation is particularly important given that Indigenous peoples comprise a substantial portion of the Circumpolar North population and their healthcare experiences are shaped by both geographic and unique cultural factors. Another gap is the disproportionate focus on evacuation studies within the field of obstetrics and gynecology. Although evacuations within this specialty can be particularly emotional and logistically challenging, it is equally important to explore evacuations in other surgical domains, such as trauma surgery and emergency/acute surgical contexts. Research in these areas will refine evacuation strategies more broadly and improve the care experiences of patients undergoing a wider range of surgical interventions. Additionally, there is a lack of patient‐centered research on receiving surgical care closer to home, beyond pre‐ and post‐operative visits and consultations. Gaining insight into patients’ experiences with telesurgery and the expansion of local surgical capacity—particularly in regions where these services have already been implemented—is a critical area for future investigation, especially given the ongoing challenges in recruiting and retaining surgical healthcare personnel in remote areas [3].

Of note, with all scoping reviews, the methodology in this study prioritizes breadth over depth and does not include a critical appraisal of study quality, which may limit the interpretability of findings and obscure important contextual nuances. Additionally, relevant literature may have been missed due to the exclusion of non‐English studies, limited database coverage, and the omission of gray literature.

## 5 Conclusion

This scoping review provides a comprehensive understanding of the unique surgical care experiences of patients from the Circumpolar North. The findings highlight a range of factors influencing care experiences, including logistical, cultural, medical, and psychosocial aspects, as well as the impact of innovative strategies to enhance local surgical care delivery. This review emphasizes the need for tailored approaches at both the individual and systemic levels—personalized strategies that address the specific needs of patients, in addition to broader structural changes aimed at improving surgical care access and delivery. These insights are essential not only for improving care for patients in the Circumpolar North, but also for advancing efforts toward more equitable healthcare systems globally. Future research should focus on addressing the gaps identified in the literature, particularly in understanding Indigenous patients' surgical care experiences and the impacts of surgical evacuations across various specialties. Such research will inform the development of more inclusive, effective strategies to improve the surgical care experience for patients from the Circumpolar North.

## Author Contributions

**Jillian Schneidman:** conceptualization, methodology, writing – original draft, formal analysis, investigation, data curation. **Sara B. A. Morel:** writing – review and editing, investigation, formal analysis. **Vanessa Ross:** writing – review and editing, investigation, methodology. **Natasha G. Caminsky:** conceptualization, methodology, writing – review and editing. **Jeremy Grushka:** writing – review and editing. **Evan G. Wong:** supervision, conceptualization, writing – review and editing, methodology.

## Conflicts of Interest

The authors declare no conflicts of interest.

## Data Availability

Data sharing not applicable to this article as no datasets were generated or analyzed during the current study.
